# Mind your head: two cases of mucosal metastasis of BRAF-mutated melanoma of the scalp

**DOI:** 10.1007/s00428-021-03120-7

**Published:** 2021-06-17

**Authors:** S. A. Koppes, A. M. R. Schrader, A. M. L. Jansen, J. A. Rijken, A. M. Kamphuis, W. A. M. Blokx

**Affiliations:** 1 Department of Pathology, University Medical Center Utrecht, Utrecht, The Netherlands; 2 Department of Pathology, Leiden University Medical Center, Leiden, The Netherlands; 3 Department of Head and Neck Surgical Oncology, University Medical Center Utrecht, Utrecht, The Netherlands; 4 Department of Medical Oncology, University Medical Center Utrecht Cancer Center, Utrecht, The Netherlands

**Keywords:** Melanoma, Mucosal melanoma, BRAF mutations, Oncologic genetics, Metastatic melanoma

## Abstract

Mucosal melanomas are rare and only a small portion bear *BRAF* mutations while cutaneous melanomas have a much higher prevalence and often harbor *BRAF* mutations. We present two cases in which, after a malignant melanocytic mucosal lesion with a *BRAF* mutation was found, the primary cutaneous source was identified and clonality confirmed between the lesions. In both cases, primary lesions occurred on the scalp, an often-overlooked site. Both lesions showed signs of regression implying that in due time these lesions could have been fully regressed and might never have been detected. In that case, the metastatic mucosal lesion would erroneously be identified as a *BRAF*-mutated mucosal melanoma. These cases give warrant; a careful dermatological inspection should be instigated when confronted with a *BRAF*-mutated mucosal melanoma. We hypothesize that some *BRAF*-mutated mucosal melanomas might actually represent metastases of regressed cutaneous melanomas.

## Introduction

Mucosal melanoma is a rare variant of melanoma that occurs primarily in a mucosal site. Patients often present in a late stage of disease and survival is poor [1, 2]. Distinguishing primary mucosal melanoma from metastatic cutaneous melanoma to a mucosal site based on histology alone can be hard, if not impossible. Molecular analysis might help to differentiate as mucosal melanomas frequently harbor *KIT* or *NRAS* mutations and only rarely *BRAF* mutations, while *BRAF* mutations are common in primary cutaneous melanomas [3, 4]. Thus, when confronted with a *BRAF*-mutated mucosal melanoma, one should be wary of metastatic cutaneous melanoma and meticulously examine the skin of the patient. Herein, we report two patients with a mucosal melanoma in which a *BRAF* mutation was identified, and in whom, only after thorough examination of the skin, eventually a primary cutaneous melanoma was found. In both cases, the cutaneous lesion was located on the hair-bearing scalp and showed extensive regression.

## Case 1

**Fig. 1:**
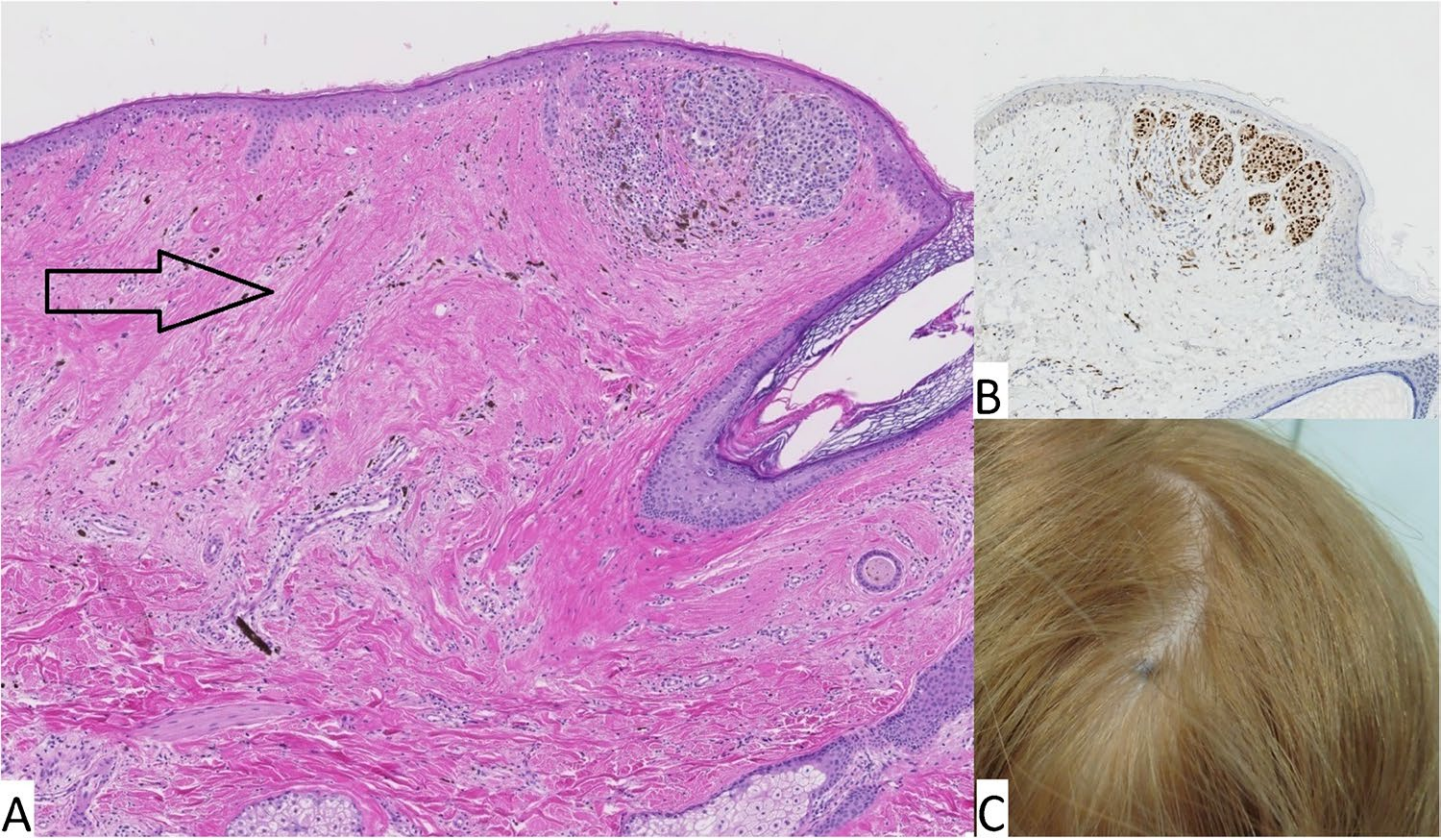
Case 1: (**A**) H&E stain of the skin lesion demonstrating a almost fully regressive melanoma; a small cluster of atypical melanocytes residing in a larger field of fibrosis (arrow) can be seen in the top right corner. These melanocytes showed strong immunohistochemical positivity for PReferentially expressed Antigen in MElanoma (PRAME, insert **B**). Macroscopically the lesion manifested as a small, slightly asymmetrical pigmented lesion on the scalp (**C**)

**Fig. 2:**
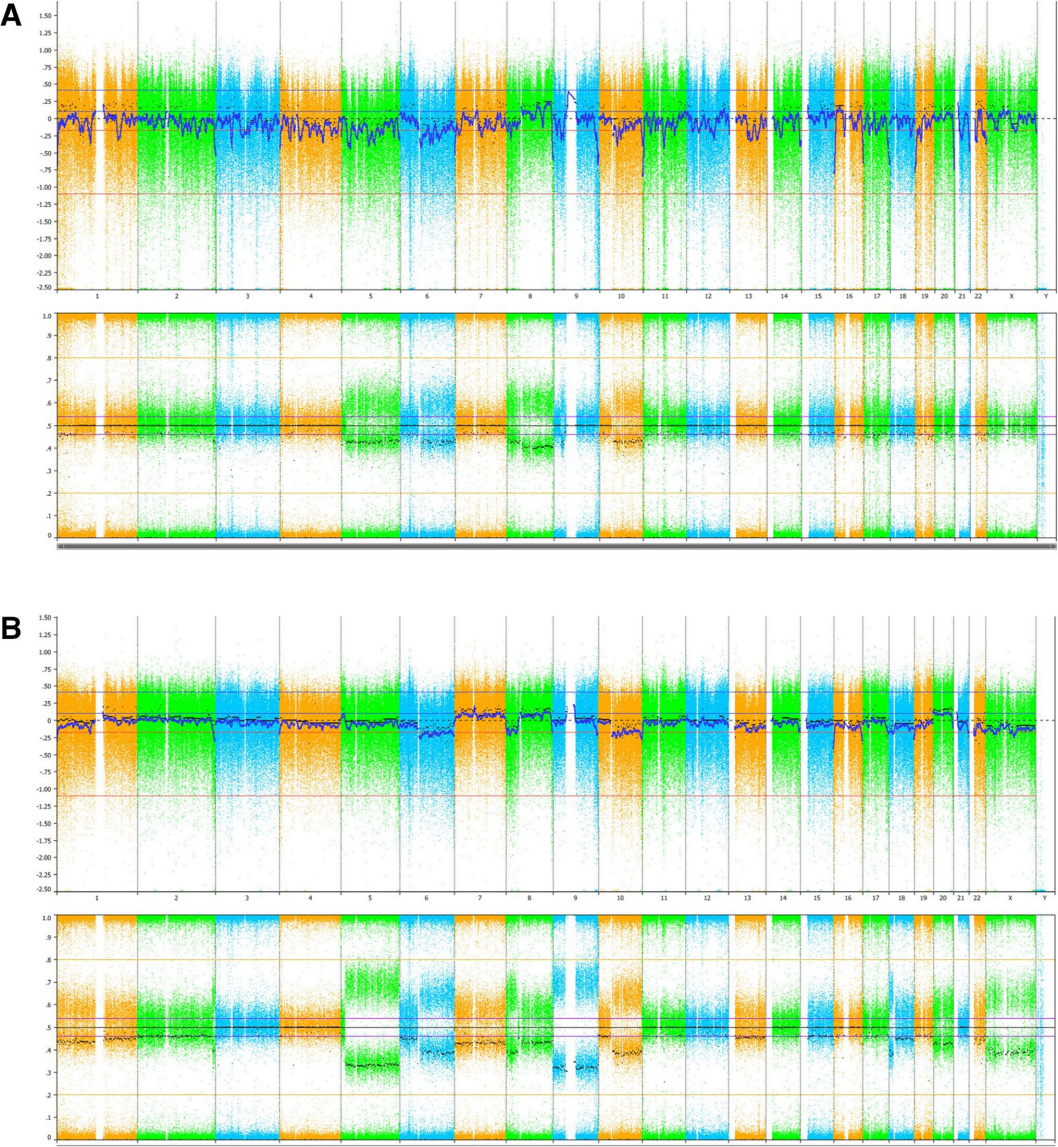
Case 1: SNP array analysis of the atypical melanocytes in the skin (**A**) and the melanoma in the maxillary sinus (**B**). **A** Upper panel shows intensity (Log R Ratio) and lower panel shows B allele frequency (BAF). CNV analysis shows partial loss of 2q, partial copy number neutral LOH (CN-LOH) of chromosome 5, loss of chromosome 6q and 10q, CN-LOH of chromosome 8p, gain of chromosome 8q, and heterozygous loss of 9p21 (including CDKN2A). **B** Upper panel shows intensity (Log R Ratio) and lower panel shows B allele frequency (BAF). CNV analysis shows CN-LOH of chromosome 1p, partial loss of chromosome 2q, partial CN-LOH of chromosome 5, loss of chromosome 6q, 8p, 10q, and 18p, trisomy of chromosome 7 and 20, gain of chromosome 8q, CN-LOH of chromosome 9, and monosomy of chromosome X

A 32-year-old female, with no prior medical history, presented with a unilateral nasal obstruction and a painful pressure-like feeling around the left eye. CT and MR imaging showed a mass in the left nasal cavity and maxillary sinus. FDG-PET scan showed multiple small lesions in both lungs and a lesion in the spine. A diagnostic nasal biopsy was performed. Microscopically the tissue comprised of mainly atypical cells with enlarged anisomorphic nuclei and focally shattered melanin pigment. The lesion showed S100 and SOX-10 expression and Melan-A (faintly). A melanoma diagnosis—either primary mucosal or metastatic—was made. Next-generation sequencing was performed which revealed a *BRAF* mutation (p.V600E) and a *TERT* promoter mutation. Before starting with nivolumab and ipilimumab, the patients’ skin was examined in order to identify a possible primary skin melanoma. On the scalp, a small pigmented macula was identified and excised for further examination. Histological examination showed a dermal nevus with a small cluster (diameter 0.8 mm) of atypical melanocytes (see Fig. 1). This cluster resided in a field of fibrosis and unlike the pre-existing dermal nevus, showed expression of PRAME (PReferentially expressed Antigen in MElanoma). Comparison of the two lesions using a SNP array demonstrated similar copy number variation patterns with a unique and identical breakpoint in chromosome 5. The additional aberrations in the nasal melanoma are attributed to tumor progression (see Fig. 2). The lesion of the scalp was therefore regarded as the primary (almost fully regressive) melanoma with metastases to the maxillary sinus and presumably also to the lungs and spine. In the first 6 weeks of treatment with nivolumab and ipilimumab, the patient showed fast tumor progression and therapy was switched to BRAF-MEK inhibition. This resulted in complete remission of the ossal metastasis in the spine and stable disease in the other sites. To achieve long term survival, the therapy was switched back to nivolumab and ipilimumab, with near complete response.

## Case 2

**Fig. 3:**
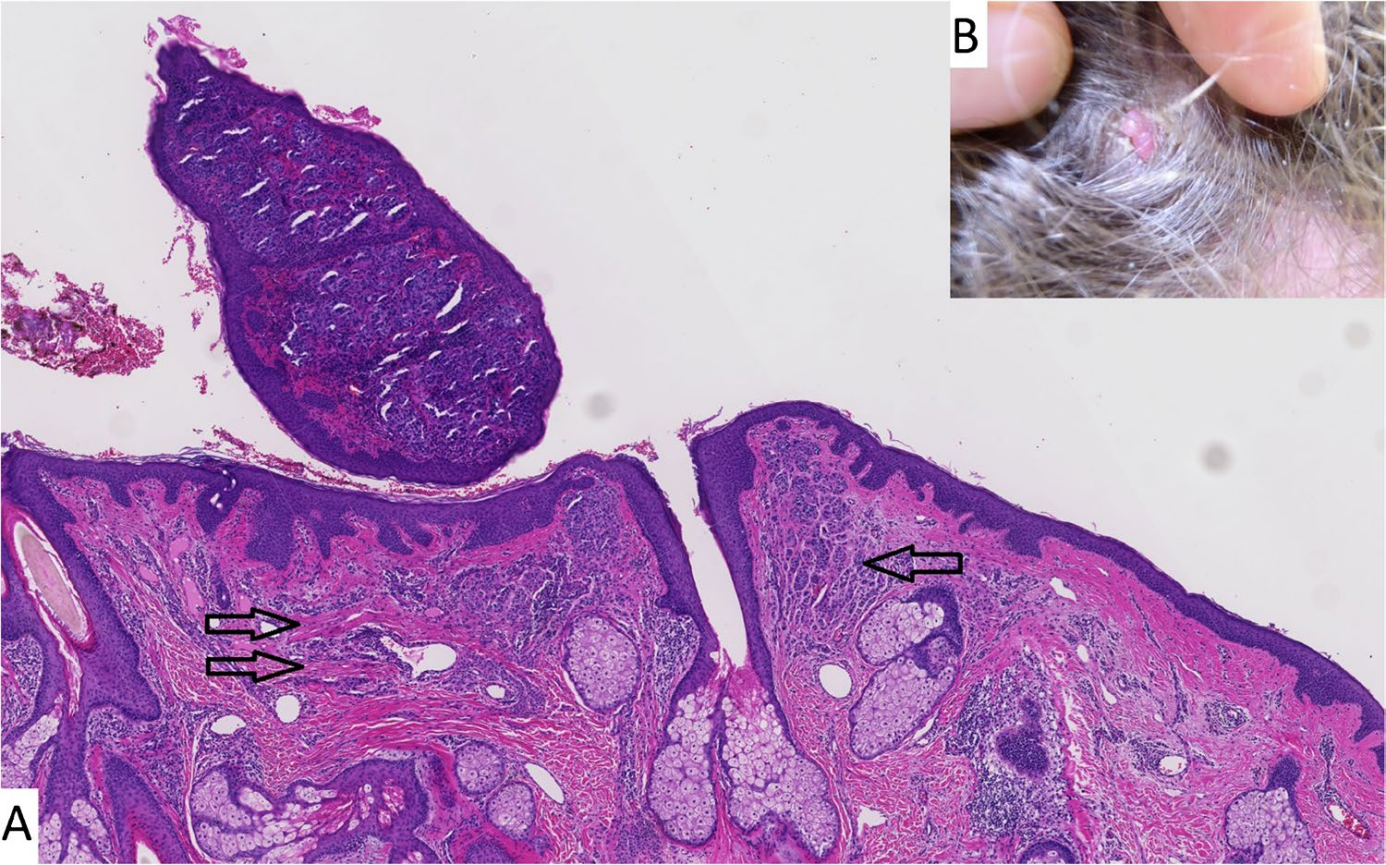
Case 2: (**A**) H&E stain of a pedunculated melanoma with regressive changes. A pre-existing dermal nevus can be seen (single arrow) and under on the right can be seen and under the pedunculated lesion regressional changes are observed (double arrow). Macroscopically the lesion manifested on scalp, clinically appearing like an angioma (**B**)

A 32-year-old male underwent a polypectomy of a polypoid lesion of the esophagus, clinically suspect for a carcinoma. On microscopy, the mucosa and submucosa showed a highly cellular proliferation of epithelioid cells with cytonuclear atypia with a varying amount of pigment and a high mitotic index. The surface was largely ulcerated but also demonstrated an intra-epithelial component consisting of irregular nests and spread of atypical cells. The morphology and immune phenotype of the tumor cells (positive for Melan-A and S100) fitted with a diagnosis of a melanoma, either primary mucosal or metastatic. Next-generation sequencing revealed the presence of the pathogenic *BRAF* p.V600E mutation, as well as likely pathogenic mutations in *CDKN2A* (p.P114L), and in the promotor region of *TERT* (C228T). PET-CT and MRI showed multiple small brain metastases. Because of the *BRAF* mutation, the skin of the patient was thoroughly examined and a small erythematous papule on the scalp, clinically appearing as an angioma, was identified (see Fig. 3B). Histology of this lesion, however, demonstrated an exophytic nodule, adjacent to a pre-existing nevus, consisting of the same atypical, epithelioid cell population as was seen in the esophageal polyp. In addition, some stromal regression was seen (see Fig. 3A). Molecular analysis demonstrated the same mutations in *BRAF*, *TERT*, and *CDKN2A*, confirming a clonal relationship between the two lesions. A *CDKN2A* germline mutation was excluded. The patient was finally diagnosed with a cutaneous nodular melanoma with esophageal and brain metastases. After 3 months of treatment with ipilimumab and nivolumab, he had progressive disease and BRAF-MEK inhibition was started, but without success. Thirteen months after initial diagnosis, the patient died of metastatic melanoma.

## Discussion

In this report, we describe two patients with a malignant melanocytic lesion in a mucosal site in which *BRAF* p.V600E mutations were identified. Approximately 50% of the patients with cutaneous melanoma harbor *BRAF* mutations, while these are rare in mucosal melanoma. Curtin et al. reported *BRAF* mutations in only 3% of 38 patients with mucosal melanomas, and Beadling et al. could not detect any *BRAF* mutation in a study of 47 patients with mucosal melanoma [5, 6]. Thus, when a *BRAF* mutation is identified in a malignant melanocytic mucosal lesion, one should always consider metastatic cutaneous melanoma.

As the two cases illustrate, identification of *BRAF* mutations not only offered our patients a systemic treatment option, but also initiated the search and identification of the primary (cutaneous) melanoma. In both cases, close examination of the patients’ skin and thorough histological and immunohistochemistry investigation identified the primary melanoma on the hair-bearing scalp, a site which is hard to inspect and easily overlooked in general dermatological inspection. In addition, in both cases, the primary skin lesions showed clear histological signs of regression. Regression is a frequently encountered phenomenon in cutaneous melanoma and some studies showed a higher risk of lymph node and visceral metastasis in lesions with regression [7–9]. Identification of the primary site is of vital importance as it can influence treatment decisions; treatment of a patient with a primary mucosal melanoma without distant metastases often consists of surgery while systemic therapy should be considered for a patient with metastatic cutaneous melanoma [10]. We hypothesize that *BRAF*-mutated mucosal melanomas, which are often encountered at a late disease stage, could actually represent metastasis of fully regressed or never-detected cutaneous melanomas. Due to the time-delay, the initial cutaneous location might long be fully regressed and clinically undetectable, a phenomenon described in metastatic cutaneous melanoma [11]. This hypothesis should be the subject of further research as this cannot be concluded on the basis of these two cases. The primary melanoma in our first patient was almost fully regressed, could only be diagnosed after multiple sections, and measured only 0.8 mm in diameter. The cutaneous lesion in our second patient was only removed because of the *BRAF* mutation in the patient’s mucosal melanoma instigated thorough dermatological investigation. Both primary lesions could easily have been missed and thus the mucosal lesion would erroneously be regarded as a primary *BRAF*-mutated mucosal melanoma.

In short, these cases teach us that *BRAF* mutations in malignant melanocytic mucosal lesions should instigate thorough clinical and histopathological investigation of skin lesions as primary lesions can easily be overlooked. In addition, one should remain critical when a malignant melanocytic mucosal lesion harbors a *BRAF* mutation and no primary skin lesion can be found as the primary cutaneous lesions can undergo complete regression and could therefore remain undetectable.
